# Supplementary material for: Content-rich biological network constructed by mining PubMed abstracts
Source: BMC Bioinformatics. 2004 Oct 8;5:147. doi: 10.1186/1471-2105-5-147 (PMC528731; doi:10.1186/1471-2105-5-147)
Supplement: Additional File 2 — The original results of the above study (non-essential files are deleted to keep the file size under the limit set by BMC bioinformatics). [file 1471-2105-5-147-S2.bz2 › chilibotAdditionalFile2/dip10/560ID9537587E10921/html/HGFAC_MET.html]

 


 **HGFAC** and **MET** 
  
Found 18 abstracts in PubMed, retrieved 10.  
 

 What does Google say? 
 PDF only 
| .edu only 

---

**Interactive relationship** (e.g. stimulation, inhibition, etc)

**Inhibitory relationship**- Tissue microarray analysis of hepatocyte growth factor  **Met**  pathway components reveals a role for  **Met** , matriptase, and hepatocyte growth factor activator  [ **HGFAC** ]  inhibitor 1 in the progression of node negative breast cancer.  Ref: 12615728 Cancer Res, 2003
- In tumorigenesis and tissue regeneration, HAI 1 regulates the hepatocyte growth factor HGF c  **Met**   [ **MET** ]  pathway by inhibiting the activity of HGF activator  [ **HGFAC** ]  HGFA  [ **HGFAC** ]  and matriptase, two serine proteases that convert pro HGF into its biologically active form.  Ref: 12815039 J Biol Chem< MedlineTA>J Biol Chem, 2003
**Neutral relationship**- CONCLUSION HGFA  [ **HGFAC** ] , HGF, and c  **Met**   [ **MET** ]  mRNA are expressed in synovial tissue in RA and OA, and HGF is activated by HGFA  [ **HGFAC** ]  and binds to c  **Met**   [ **MET** ]  on endothelial cells, inducing angiogenesis.  Ref: 11508578 J Rheumatol, 2001

**Non-interactive relationship** (e.g. studied together, co-existance, homology, etc.)

- The highly invasive breast cancer cells MDA MB 231 expressed large amounts of both c  **Met**   [ **MET** ]  and HGFA  [ **HGFAC** ] , to enable maximum influence from HGF SF and did not express the HAI 1 gene at all, which suggests a shift in the activation inhibition balance to enhance metastatic potential.  Ref: 11562767 Int J Oncol, 2001
- Its actions are dependent on activation by HGF activator  [ **HGFAC** ]  HGFA  [ **HGFAC** ]  and its binding to a specific HGF receptor  [ **MET** ]  c  **Met**   [ **MET** ] .  Ref: 11508578 J Rheumatol, 2001
- The balance between HGF SF activation and HGFA  [ **HGFAC** ]  inhibition is critical to the metastatic potential of the tumour cells, and the invasive nature of the cancer cell lines correlated to the degree of c  **Met**   [ **MET** ]  and or HGFA  [ **HGFAC** ]  presence along with HAI 1 expression.  Ref: 11562767 Int J Oncol, 2001
- RT PCR showed HGF, HGFA  [ **HGFAC** ] , and c  **Met**   [ **MET** ]  mRNA in RA, OA, and control synovial tissue.  Ref: 11508578 J Rheumatol, 2001
- In contrast, the breast cancer cells of low invasive nature MCF 7 displayed a low level of c  **Met**   [ **MET** ]  and HGFA  [ **HGFAC** ]  expression, while expressing the HAI genes to a high degree.  Ref: 11562767 Int J Oncol, 2001
- The cell lines all displayed different patterns of expression, and in some of the cancer cell lines the concomitant expression of the HGF SF, c  **Met**   [ **MET** ] , HGFA  [ **HGFAC** ]  and HAI genes was observed.  Ref: 11562767 Int J Oncol, 2001
- The present study examined the expression of HGF SF, HGFA  [ **HGFAC** ] , the two inhibitors to HGFA  [ **HGFAC** ]  action known as hepatocyte growth factor activator  [ **HGFAC** ]  inhibitors type 1 and 2 HAI 1 and HAI 2, and the HGF SF receptor  [ **MET** ] , c  **Met**   [ **MET** ] .  Ref: 11562767 Int J Oncol, 2001
- The only cell line to produce a significant amount of HGF SF was the human fibroblasts MRC 5 which also co expressed the c  **Met**   [ **MET** ]  and HGFA  [ **HGFAC** ]  genes to allow autocrine regulation of HGF SF stimulation, and importantly displayed little or no inhibitor presence to suppress the biological function of HGF SF.  Ref: 11562767 Int J Oncol, 2001
- The most prominent differences between advanced and local adenocarcinoma were upregulation of COL3A1, CFGR, and  **MET**  in advanced carcinoma, and downregulation of  **HGFAC** , FZD3, and BFL1 in the same tumors.  Ref: 11454421 Cancer Genet Cytogenet, 2001
- ISH and immunohistochemistry revealed mRNA expression for HGF, HGFA  [ **HGFAC** ] , and c  **Met**   [ **MET** ]  in the cell types mentioned above.  Ref: 11508578 J Rheumatol, 2001
- Other changes in carcinoma were upregulation of  **MET**  and Ne dlg, and downregulation of  **HGFAC** , desmin, and PDGFA.  Ref: 11454421 Cancer Genet Cytogenet, 2001
- The gene expression of HGF and keratinocyte growth factor KGF in gingival connective tissue, and c  **met**   [ **MET** ] , HGFA  [ **HGFAC** ]  and KGF receptor KGFR in gingival epithelial tissues was monitored using RT PCR.  Ref: 12613501 J Oral Sci, 2002
- Although the presence of hepatocyte growth factor and its c  **MET**   [ **MET** ]  receptor have been reported in male mammalian reproductive tracts, our novel findings on the secretion of HGFA  [ **HGFAC** ]  precursor from turkeys may help to elucidate the regulation of activated hepatocyte growth factor.  Ref: 12128063 Comp Biochem Physiol B Biochem Mol Biol, 2002
- METHODS Immunohistochemical staining, Western blotting, RT PCR, and in situ hybridization ISH for HGF, HGFA  [ **HGFAC** ] , and c  **Met**   [ **MET** ]  were performed on synovial tissue specimens from 10 patients with RA and 4 with OA, and 2 healthy controls.  Ref: 11508578 J Rheumatol, 2001
- Western blot analyses using antibodies against hepatocyte growth factor, hepatocyte growth factor receptor  [ **MET** ] , phosphotyrosine, hepatocyte growth factor activator  [ **HGFAC** ] , urokinase type plasminogen activator receptor, urokinase type plasminogen activator, plasminogen activator inhibitor 1, fibrinogen and plasmin were performed on membrane enriched fractions from the frozen tissue, as was collagen zymography for matrix metalloproteinase 2 and matrix metalloproteinase 9.  Ref: 12527708 Mod Pathol, 2003
- Hepatocyte growth factor HGF, HGF activator  [ **HGFAC** ] , and c  **Met**   [ **MET** ]  in synovial tissues in rheumatoid arthritis and osteoarthritis.  Ref: 11508578 J Rheumatol, 2001
- To determine whether these factors contribute to the distinct etiologies of fibrolamellar hepatocellular carcinoma and traditional hepatocellular carcinoma, we assayed hepatocyte growth factor, the hepatocyte growth factor receptor  [ **MET** ] , and two hepatocyte growth factor activators, hepatocyte growth factor activator  [ **HGFAC** ]  and urokinase type plasminogen activator, in hepatocellular carcinoma, fibrolamellar hepatocellular carcinoma, cirrhotic liver and normal liver.  Ref: 12527708 Mod Pathol, 2003
- To examine the role of the HGF system in epithelial invasion followed by loss of connective tissue attachment in periodontitis, mRNA expression of HGF, its receptor c  **met**   [ **MET** ]  and HGFA  [ **HGFAC** ]  in gingival tissues was monitored.  Ref: 12613501 J Oral Sci, 2002
- We examined the role of HGF, HGFA  [ **HGFAC** ] , and c  **Met**   [ **MET** ]  in synovial tissues in rheumatoid arthritis RA and osteoarthritis OA, and their localization and mRNA expression.  Ref: 11508578 J Rheumatol, 2001
- RESULTS Immunohistochemical staining revealed that HGFA  [ **HGFAC** ]  and c  **Met**   [ **MET** ]  were strongly expressed in fibroblasts, macrophages, endothelial cells, and synovial lining cells.  Ref: 11508578 J Rheumatol, 2001
